# Developmental perturbation in human embryos: Clinical and biological significance learned from time‐lapse images

**DOI:** 10.1002/rmb2.12593

**Published:** 2024-07-09

**Authors:** Kenji Ezoe, Tsubasa Takahashi, Tetsuya Miki, Keiichi Kato

**Affiliations:** ^1^ Kato Ladies Clinic Tokyo Japan

**Keywords:** assisted reproductive technology, developmental competence, embryo development, embryo selection, time‐lapse imaging

## Abstract

**Background:**

Time‐lapse technology (TLT) has gained widespread adoption worldwide. In addition to facilitating the undisturbed culture of embryos, TLT offers the unique capability of continuously monitoring embryos to detect spatiotemporal changes. Although these observed phenomena play a role in optimal embryo selection/deselection, the clinical advantages of introducing TLT remain unclear. However, manual annotation of embryo perturbation could facilitate a comprehensive assessment of developmental competence. This process requires a thorough understanding of embryo observation and the biological significance associated with developmental dogma and variation. This review elucidates the typical behavior and variation of each phenomenon, exploring their clinical significance and research perspectives.

**Methods:**

The MEDLINE database was searched using PubMed for peer‐reviewed English‐language original articles concerning human embryo development.

**Main findings:**

TLT allows the observation of consecutive changes in embryo morphology, serving as potential biomarkers for embryo assessment. In assisted reproductive technology laboratories, several phenomena have not revealed their mechanism, posing difficulties such as fertilization deficiency and morula arrest.

**Conclusion:**

A profound understanding of the biological mechanisms and significance of each phenomenon is crucial. Further collaborative efforts between the clinical and molecular fields following translational studies are required to advance embryonic outcomes and assessment.

## 1 INTRODUCTION

Time‐lapse technology (TLT) uses digital cameras built into an incubator for continuous monitoring of embryos under stable and uninterrupted conditions, avoiding the need to remove embryos from the incubator for assessment. TLT was first implemented for research use to monitor human embryo kinetics, revealing the time course of fertilization and early embryo development.^1^ Subsequent studies highlighted the potential use of TLT as a clinical tool,^2^ leading to its widespread adoption worldwide.^3^, ^4^ In addition to providing an undisturbed culture environment for embryos, TLT offers the advantage of continuous monitoring, enabling the detection of spatiotemporal changes that may otherwise go unnoticed (Video S1).^5^ Several phenomena observed during fertilization, cleavage, compaction, and blastulation have been reported and reviewed, which are considered to enable optimal embryo selection and deselection, leading to improvements in assisted reproductive technology outcomes.^6^ However, the clinical advantages of introducing TLT remain unclear.

**FIGURE 1:**
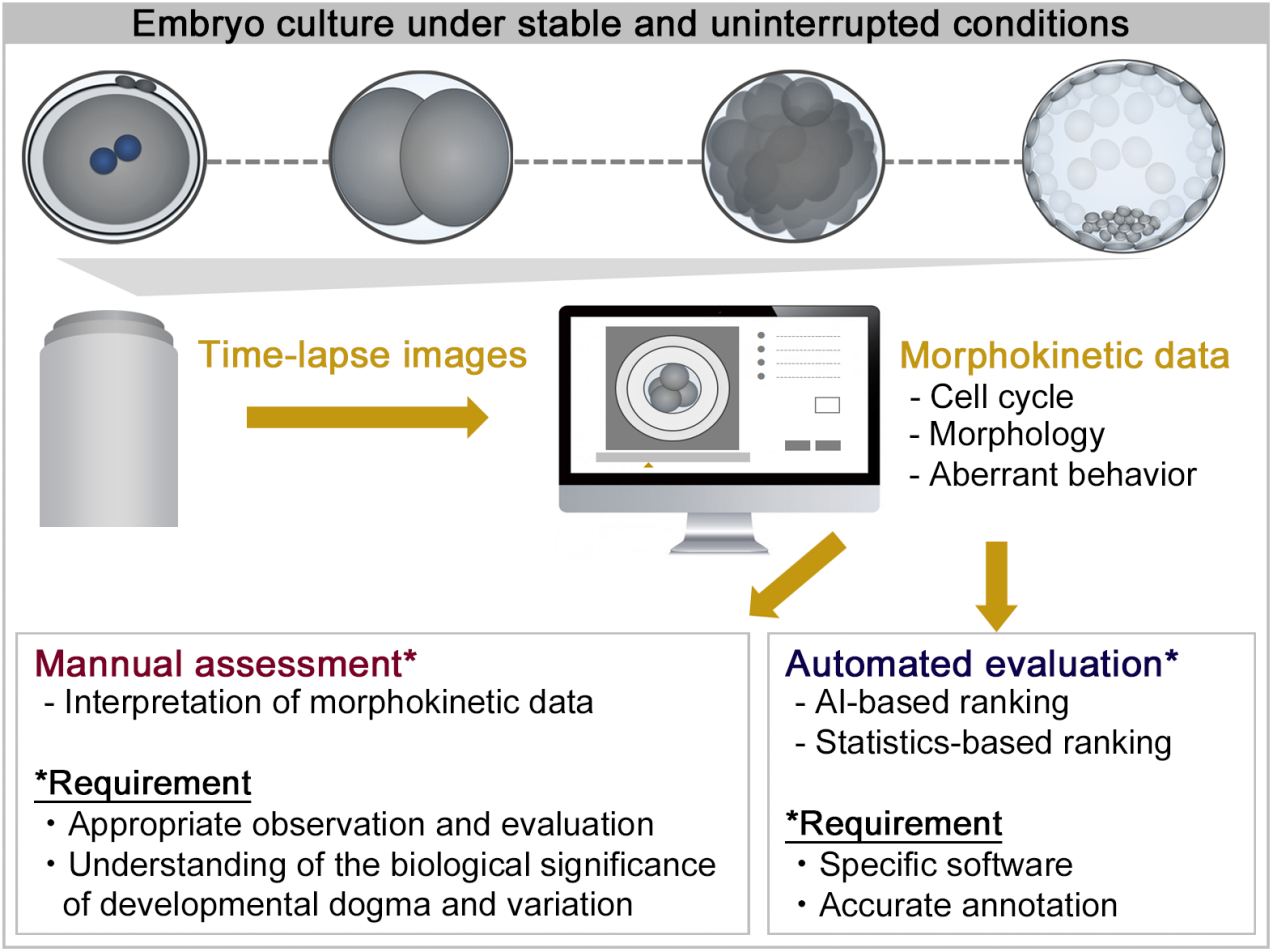
Embryo culture and ranking using time‐lapse technologies. In addition to facilitating the undisturbed culture of embryos, time‐lapse technologies offer the unique capability of continuously monitoring embryos to detect spatiotemporal changes. Combining commercial‐based, automated embryo evaluation systems with manual assessment of embryo perturbation could facilitate a detailed assessment of competence for development and subsequent implantation. However, manual annotation requires appropriate knowledge of embryo observation and an understanding of the biological significance of developmental dogma and variation. AI, artificial intelligence.

In a 2019 Cochrane database of systematic reviews, insufficient high‐quality evidence was found regarding differences in pregnancy outcomes when comparing TLT with or without embryo selection software to conventional incubation.^7^ Despite the publication of this review, the clinical benefit of implementing TLT remains controversial, with six additional randomized control trials reporting conflicting results (Table 1).^8^, ^9^, ^10^, ^11^, ^12^, ^13^ This controversy may arise from several factors. Firstly, these studies primarily utilize commercial‐based, automated embryo evaluation systems, which vary in their software implementations. Secondly, the algorithms used for embryo evaluation may not encompass all embryonic phenomena routinely considered predictive factors for embryo development and pregnancy outcomes in embryology laboratories. Lastly, the interpretation of artificial intelligence‐based evaluation systems lacks transparency, leaving uncertainty regarding whether these systems accurately identify abnormal phenomena that could impact clinical outcomes. Therefore, it is imperative to validate the clinical efficacy of these systems in each laboratory before their adoption. On the other hand, combining these automated systems with manual assessment of embryo perturbation could facilitate a detailed prediction of competence for development and subsequent implantation. Manual assessment requires appropriate knowledge of embryo evaluation and an understanding of the biological significance of developmental dogma and variation (Figure 1). In this review, we describe the typical behavior and variation of each phenomenon with videos, exploring their clinical significance and research perspectives.

**TABLE 1:** Recent randomized controlled trials assessing the clinical benefit of time‐lapse technology.

	Year	TLT incubator	Outcomes	Embryo selection	Results
Ahlstrom et al.^8^	2022	EmbryoScope	Ongoing pregnancy, early pregnancy loss	KIDScore or routine (Blastocyst)	(Not beneficial) TLT‐based selection did not improve ongoing pregnancy rates compared to morphology alone
Guo et al.^9^	2022	EmbryoScope	Clinical pregnancy, live birth, birth weight	KIDScore or routine (Cleavage)	(Beneficial) TLT has a significant benefit on clinical pregnancy rates and overall birth weights while morphokinetic analysis was shown to be unnecessary
Kermack et al.^10^	2022	EmbryoScope	Blastocyst formation, embryo metabolism	–	(Beneficial) Culturing embryos in a TLT incubator was associated with a higher Day 5 blastocyst formation rate and altered amino acid utilization
Kieslinger et al.^11^	2023	Geri	Live birth, cumulative live birth	EEVA or routine (Cleavage)	(Not beneficial) Neither TLT‐based embryo selection using the EEVA test nor uninterrupted culture conditions in a TLT incubator improved clinical outcomes compared with routine methods
Meng et al.^12^	2022	EmbryoScope	Clinical pregnancy, live birth	KIDScore or routine (Cleavage)	(Not beneficial) Elective single cleavage‐stage embryo transfer with TLT‐based selection did not have any advantages over conventional morphological evaluation
Zhang et al.^13^	2022	Geri	Implantation, live birth, cumulative live birth	Geri Assess 1.2 software or routine (Cleavage)	(Not beneficial) The implantation rate in the first embryo transfer cycle was significantly improved in the TLT group, but the effect of TLT on the live birth or cumulative live birth rate was not significant

## 2 FERTILIZATION STAGE

### 2.1 Pronuclear formation and breakdown

#### 2.1.1 Typical behavior

During fertilization, the paternal and maternal DNA undergo decondensation, forming pronuclei (PNs), a process known as “PN formation.” The male and female PNs then migrate toward the center of the ooplasm, where their envelopes interdigitate, referred to as “PN juxtaposition.” Following this, the nuclear envelope becomes indiscernible, termed “PN breakdown (PNBD).” The most common clinical practice involved simply counting the number of PNs. Zygotes with two PNs were identified as normally fertilized zygotes that were available. Discrimination between female and male PNs is based on mutual positions relative to the second polar body (female) and positional association with the cytoplasmic wave (male). The latter is interpreted as the morphokinetic manifestation of the microtubule aster radiation organized by sperm centrioles.^1^, ^14^ Male and female PNs typically emerged almost simultaneously at approximately 5–6 h post‐insemination (hpi) and juxtaposed at 8–10 hpi.^14^, ^15^, ^16^ Prior to PNBD, the area of female and male PNs were approximately 530 and 610 μm^2^, respectively.^16^ The breakdown of female and male PNs occurs concurrently at 23–25 hpi,^14^, ^17^, ^18^, ^19^ with a time interval (TI) from PNs appearance to PNBD lasting approximately 17–19 h (Table 2).

**TABLE 2:** Developmental variation during fertilization.

Phenomena	Variation	Videos	Outcomes
PN growth	Unequal‐sized PNs	Video S2	Embryo quality, live birth
PNBD	Delayed PNBD	Video S3	Embryo quality, implantation, live birth
	Early PNBD	Video S4	Missing PNs at fertilization check in static observation
	Non‐juxtaposed PNBD	Video S5	Abnormal cleavage, blastocyst formation
	Asynchronous PNBD	Video S6	Abnormal cleavage, blastocyst formation
NPB alignment	NPB misalignment	Video S7	Controversial
	NPB alignment in a1PN zygote	Video S8	–
	NPB alignment in a non‐juxtaposed zygote	Video S9	–
Cytoplasmic halo	Absence of a cytoplasmic halo	Video S10	Abnormal cleavage, blastocyst formation
	Prolonged cytoplasmic halo	Video S11	Abnormal cleavage, blastocyst formation, ongoing pregnancy
	Unstable cytoplasmic halo	Video S12	–

Abbreviations: NPB, nucleolus precursor body; PN, Pronuclear/pronucleus; PNBD, pronuclear breakdown.

#### 2.1.2 Variation and clinical significance

The size and growth pattern of PNs vary among embryos (Video S2). A smaller difference in areas between male and female PNs immediately before PNBD (approximately < 40 μm^2^) was positively associated with embryo quality and live birth.^20^, ^21^ Furthermore, the difference between each PN size 8 h before PNBD should be larger than the difference in size immediately before PNBD. Embryos meeting these criteria are recommended for transplantation, while pregnancies were not achieved when the female PN was larger than the male PN in size.^21^

The timing of PNBD and the duration of the PN stage varies among embryos (Video S3). The delay in PNBD (>25 h) and prolonged TI from PN appearance to PNBD (>20 h) are associated with poor embryo quality on day 3, affecting implantation and live birth outcome, although the optimal range or cut‐off value have not been reported.^14^, ^17^, ^18^, ^19^, ^22^ Therefore, embryos without these phenomena should be prioritized for transfer. Furthermore, the proportion of oocytes with visible PNs was highest at 16–16.5 hpi (98.3%).^23^ At 18–18.5 h post‐insemination, the number of visible PNs reduces to 95.5% and further declines to 87.0% at 19.5–20 hpi. This implies that the optimum time to perform fertilization assessment for oocytes cultured in standard incubation is 16.5 ± 0.5 h post‐insemination (Video S4). Therefore, the current consensus recommends fertilization verification at 16–18 hpi,^24^, ^25^ and requires modification to minimize the chance of fertilization being missed, otherwise known as “0PN” zygotes.^23^, ^26^, ^27^, ^28^

Some zygotes (2%–5%) exhibit non‐juxtaposed PNBD (Video S5), with a PNs distance of 8.7 ± 0.7 μm (range, 3.5–18.9 μm).^16^, ^29^ Non‐juxtaposition of PNs is associated with an increased incidence of direct cleavage in the first mitosis and subsequent decreased blastocyst development. However, it does not affect pregnancy outcomes after blastocyst transfer.^29^ Consequently, these zygotes should be cultured until the blastocyst stage is attained.

**FIGURE 2:**
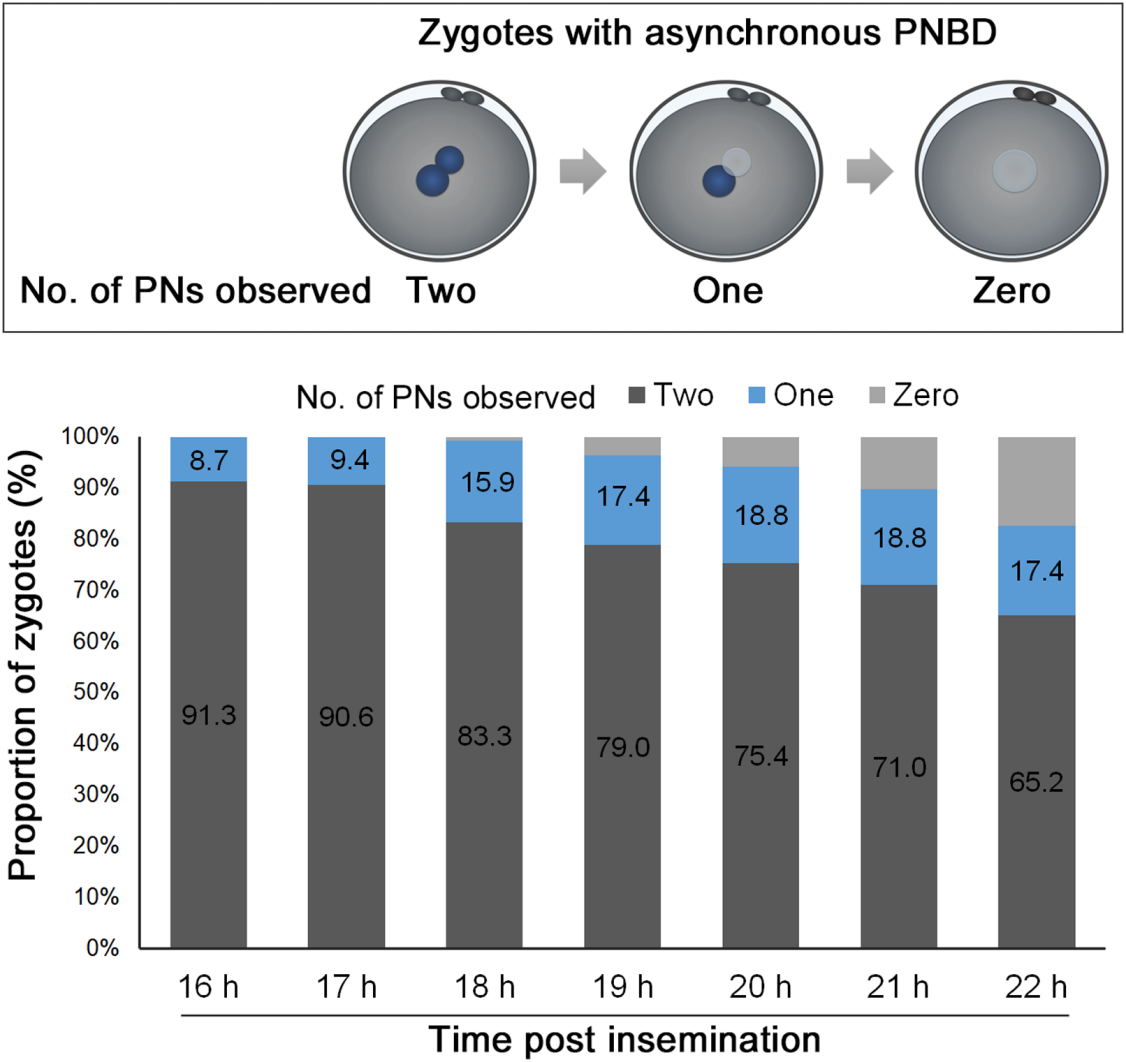
The number of pronuclei observed during fertilization verification at 16–22 hpi in zygotes with asynchronous pronuclear breakdown. PNs, pronuclei.

Furthermore, a minority of zygotes (1%–2%) demonstrate asynchronous PNBD (Video S6), with a TI of 3.9 ± 1.5 h (range, 0.5–20.1 h) between each PNBD event.^16^, ^29^ Asynchronous PNBD is a cause of 1PN zygotes found during static observation. In these zygotes, one PN had already disappeared by the time of fertilization verification at 16–22 hpi, although two PNs were present after insemination (Figure 2). Asynchronous PNBD is likely to increase rapid cleavage during the first mitosis and decrease blastocyst development. However, no adverse effects on pregnancy after blastocyst transfer have been observed.^29^ Therefore, these embryos are preferred for culturing until the blastocyst stage.

#### 2.1.3 Current and future research perspectives

Although the association of PN behavior and morphology with various fertility endpoints has been extensively examined and reviewed,^6^, ^30^, ^31^, ^32^ the mechanism regulating PN dynamics remains enigmatic. Nuclear size reportedly depends on the chromosome content,^33^ the ratio between the amount of chromatin organized in the PN and the cytoplasm volume,^34^ and the quantity of zygotic nuclear filamentous actin.^35^ Furthermore, female PN size decreases with maternal aging, whereas male PN size remains unaffected.^15^ In addition, both female and male PN were larger in 1PN zygotes and smaller in 3PN zygotes than in 2PN zygotes.^16^ However, the precise mechanisms underlying the regulation of PN size and growth remain unclear.

PN migration and juxtaposition are regulated by microtubules, dynein, and centrosomes,^36^ suggesting that the non‐juxtaposition and displacement of PNs may be induced by disrupting this regulatory system. However, the factors causing disturbances in these systems remain unknown.

Asynchronous PNBD is typically linked to asynchronous nucleolus precursor body (NPB) alignment and errors in chromosome capture, resulting in chromosome segregation errors and micronuclei formation at the 2‐cell stage.^36^ Furthermore, maternal aging increases the incidence of asynchronous PNBD.^15^ Further studies are required to reveal the mechanisms by which maternal aging affects PNBD synchrony.

### 2.2 NPB alignment

#### 2.2.1 Typical behavior

The nucleoli in oocytes and zygotes are commonly referred to as NPBs. NPBs, which exhibit compact structure and morphological distinction from nucleoli in somatic cells,^37^ move to the area of PN juxtaposition, known as “NPB alignment.” The clustering and alignment of NPBs mirrored the distribution of PN chromatin. As the PN envelopes dissolve in preparation for the first mitosis, this chromatin arrangement plays a crucial role in facilitating the recruitment of chromosomes by kinetochore fibers of the mitotic spindle and their proper arrangement in the metaphase plate. Therefore, the non‐invasive NPB observation provides reliable information on chromatin distribution.^36^ The polarization of NPBs occurs at the interface of the PN juxtaposed area, with female PN exhibiting this phenomenon earlier (8–13 hpi) than male PN (11–17 hpi).^14^, ^15^, ^16^

#### 2.2.2 Variation and clinical significance

Some zygotes do not exhibit NPB alignment (Video S7). Moreover, the incidence of alignment varies between female (approximately 65%–75%) and male (approximately 40%–50%) PNs.^15^, ^16^ Although the large difference in the number of NPBs in both PNs and NPB non‐alignment is reportedly associated with decreased competence for the preimplantation development, the influence of NPB alignment on pregnancy outcomes, including clinical pregnancy and implantation, remains controversial.^38^, ^39^, ^40^, ^41^

NPB migration speed differs among zygotes. It is positively associated with the rates of euploid blastocysts and live birth, with a cut‐off value of approximately 3.7–4.6 μm/h.^42^, ^43^ Measuring the speed by specific software may serve as a predictor of embryonic and pregnancy outcomes.

#### 2.2.3 Current and future research perspectives

The NPB alignment is observed in both maternally and paternally derived 1PN zygotes (Video S8) and non‐juxtaposed PN zygotes (Video S9), suggesting that NPB alignment is not necessarily associated with PN juxtaposition.^16^, ^29^ However, it remains unclear why NPB alignment with the interface of the PN juxtaposition does not occur in some embryos. Recent high‐resolution live cell imaging of bovine and human zygotes revealed that in the process of PN migration and chromatin clustering, dynein links to nuclear pore complexes and transports the PNs along centrosome‐nucleated microtubules, establishing PN juxtaposition.^36^ While two PNs are pulled into proximity, the nuclear pore complexes and parental genomes also migrate and polarize at the interface of the juxtaposition. Therefore, the clustering and polarization of parental genomes toward each other, reflected by the NPB alignment, are considered to be driven by dynein, microtubules, and nuclear pore complexes. Thus, it is hypothesized that a deficiency in these systems may cause a failure in NPB alignment. Further studies are required to elucidate the mechanism of this phenomenon and to develop a treatment that can prevent misalignment.

### 2.3 Cytoplasmic halo

#### 2.3.1 Typical behavior

“Cytoplasmic halo,” which involves the centripetal redistribution of cytoplasmic granules and organelles, generating a translucent moon‐shaped cytoplasmic domain in the cortex, occurs during human fertilization.^1^, ^44^ Cytoplasmic granules initiate movement toward the central ooplasm, with peripheral translucency first confirmed (translucent halo appearance, tHa) at 8 hours post‐insemination (hpi).^45^ This centripetal movement halts (cytoplasmic granule condensation, tHc) until 13 hpi. Subsequently, granules return to the cortex (cytoplasmic granule redistribution, tHr) at 20 hpi, completing the redistribution (translucent halo disappearance, tHd) at 22 hpi, just before PNBD. The total duration of the cytoplasmic halo is about 14–15 h.^45^ This phenomenon is observed in approximately 80%–95% of normally fertilized oocytes.^14^, ^45^, ^46^, ^47^ However, it is absent in non‐inseminated or non‐fertilized oocytes and detectable in abnormally fertilized oocytes.^16^, ^30^

#### 2.3.2 Variation and clinical significance

The absence of a cytoplasmic halo is associated with significantly higher rates of abnormal cleavage (rapid, reverse, or asymmetric cleavage) and impairment in blastocyst formation (Video S10).^45^ The embryo development to the blastocyst stage and pregnancy outcomes after cleavage‐stage embryo transfers are adversely affected by the halo absence^45^, ^46^, ^48^; however, pregnancy rates after blastocyst transfers are comparable between embryos derived from halo‐positive zygotes and embryos derived from halo‐negative zygotes.^45^ Therefore, zygotes without the cytoplasmic halo should be cultured to the blastocyst stage. Categorizing the halo as symmetrical or asymmetrical depends on its position relative to the cell center^47^; the distribution of the halo is not associated with developmental and pregnancy outcomes.^45^, ^47^ Therefore, the observation of halo distribution is not required.

Prolonged halo adversely affects preimplantation development and pregnancy outcomes (Video S11).^14^, ^45^ Therefore, based on our preliminary data, embryos exhibiting appropriate cytoplasmic halo duration, a TI from translucent halo appearance (tHa) to translucent halo disappearance (tHd) of less than 16 h, and a TI from cytoplasmic granul condensation to cytoplasmic granul redistribution of less than 10 h should be prioritized for transfer, especially in cases where multiple transferable embryos are available.

#### 2.3.3 Current and future research perspectives

The cytoplasmic halo serves as a marker for the relocation of mitochondria and other cytoplasmic components from the cell periphery to the center to support PN function.^30^, ^49^ Although similar organelle clustering has been observed in other species, the mechanism and biological significance of this halo remain uncertain.^50^ Furthermore, the characteristics of the cytoplasmic halo are strongly associated with patient and gamete characteristics, such as male age, oocyte diameter, and sperm quality^45^, ^46^; therefore, these factors could influence the regulation of microtubule‐organized translocation in the ooplasm during fertilization. Interestingly, a minority of zygotes (1.6%) exhibit continuous movement of cytoplasmic granules, and the peripheral halo wavers until the first cell division (Video S12).^45^ As PNs migrate toward the central ooplasm and align, the formation of the cytoplasmic halo occurs, suggesting the involvement of molecular actors like dynein and microtubules^36^ in orchestrating the centripetal redistribution of cytoplasmic granules and organelles. However, it's worth noting that the centripetal movement and juxtaposition of PNs can occur even in zygotes lacking a visible cytoplasmic halo.^16^ To elucidate the factors governing granule and organelle movement and understand the variability in cytoplasmic halo presence and duration, further investigations utilizing knockout or conditional knockout animal embryo models are warranted.

## 3 CLEAVAGE STAGE

### 3.1 First cell division (first mitotic division/first cleavage)

#### 3.1.1 Typical behavior

After PNBD, the chromosomes of both gametes arrange themselves on the spindle, mediating mitosis, known as “first cell division.” Two equivalent blastomeres were typically generated after the first cell division occurring at 24–28 hpi.^14^, ^16^, ^51^, ^52^ The TI from PNBD to the first cell division is 2–3 h,^53^ and the duration of the first cytokinesis is 0.2–0.3 h^19^, ^54^ (Table 3).

**TABLE 3:** Developmental variation during the cleavage stage.

Phenomena	Variation	Videos	Outcomes
First cell division	Direct cleavage	Video S13	Blastocyst formation
	Rapid cleavage	Video S14	Blastocyst formation
	Asymmetric division	Video S15	–
	Reverse cleavage	Video S16	Early cleavage, implantation
Blastomere movement	Blastomere wobbling	Video S17	No impact
	Twist‐and‐crumble	Video S18	Blastocyst formation
	Prolonged blastomere movement	Video S19	Blastocyst formation
Transzonal projection loss	Perivitelline threads	Video S20	Fragmentation
Nucleation	Multinucleation	Video S21	Controversial

#### 3.1.2 Variation and clinical significance

The delay in the first cell division is associated with a decreased developmental rate, poor embryo quality, and adverse pregnancy outcomes.^14^, ^22^, ^51^, ^52^ Zygotes with the first cell division occurring after 28 hpi likely have a lower chance of blastocyst formation and pregnancy.^51^, ^52^

Direct cleavage (Video S13) and rapid cleavage (Video S14): the phenomenon by which an embryonic cell divides into three daughter blastomeres via single multichotomous mitosis (duration of 2‐cell stage = 0 h) is termed “direct cleavage”.^55^ The phenomenon where two consecutive mitoses are separated by a very short intervening time (duration of 2‐cell stage >0 h and <5 h) is termed “rapid cleavage”.^6^, ^56^, ^57^ It is essential to distinguish between these phenomena at the blastomere level and large‐generated fragmentation. The incidences of direct and rapid cleavage are 4%–6% and 7%–12%, respectively.^15^, ^16^ Embryos with direct and rapid cleavage exhibit lower rates of blastocyst formation and pregnancy after cleavage‐stage embryo transfers compared to embryos with normal cleavage.^56^, ^57^, ^58^ The influence of direct and rapid cleavage on pregnancy outcomes after blastocyst transfers has been controversial.^57^, ^59^, ^60^, ^61^ However, a recent large cohort study concluded that direct cleavage does not impact the live birth rate once the embryo develops to the blastocyst stage.^58^ Therefore, the deselection of the embryos for transfer at the cleavage stage is recommended.

In asymmetric division (Video S15), two uneven‐sized blastomeres are generated after the first cell division (incidence, 20%–25%).^15^, ^16^ Although the clinical significance of this phenomenon observed using the TLT has not been reported, this phenomenon can be considered an atypical behavior suggested by the association with the absence of a cytoplasmic halo.^45^

Reverse cleavage (Video S16) is the phenomenon where two cells fuse into one blastomere (also called cell fusion).^6^, ^56^ This phenomenon must be distinguished from fragment internalization or reabsorption through the identification of a nucleus within the cells. The incidence of reverse cleavage during the first cell division is 5%–8%.^15^, ^16^, ^62^ Embryos showing reverse cleavage are less competent during embryonic development and pregnancy.^61^, ^63^, ^64^ Therefore, embryos that do not exhibit this phenomenon should be prioritized for transfer.

#### 3.1.3 Current and future research perspectives

As previously mentioned, the mechanism of abnormal cleavage during the first cell division has not been completely elucidated. As reviewed by Coticchio et al.,^65^ the formation of multipolar spindles is postulated to trigger multichotomous cleavage, which is expected to unevenly distribute chromosomes in the three daughter blastomeres, thereby creating a chaotic aneuploidy.^66^, ^67^, ^68^ Following multipolar zygotic division, fewer embryos reach the blastocyst stage, and diploidization occurs frequently, indicating that blastomeres with genome‐wide errors resulting from whole‐genome segregation errors can either be selected against or contribute to embryonic arrest.^69^, ^70^, ^71^ Regarding rapid cleavage, a short cell cycle may be insufficient to allow complete DNA replication and repair prior to chromosomal alignment.^57^ Depending on the timing of reverse cleavage, this phenomenon can result in aneuploidy or polyploidy.^32^ However, the mechanisms underlying the rapid and reverse cleavage remain unclear.

### 3.2 Blastomere movement

#### 3.2.1 Typical behavior

During culture in a time‐lapse system, immediately after the first mitosis, the cell membrane and cytoplasm of the blastomeres move in several directions during the 2‐cell stage, known as “blastomere movement”.^72^ Approximately half of the embryos experience transient shrinking and expansion, which is categorized as “bouncing” and lasts 2–3 h.

#### 3.2.2 Variation and clinical significance

Approximately 20% of embryos exhibit continuous cytoplasmic and membrane waving, defined as “wobbling” (Video S17).^72^, ^73^, ^74^ About 30% exhibit “twist‐and‐crumble” type, involving blastomere rolling, followed by fragment generation (Video S18).^72^, ^75^ The development rates of wobbling embryos were comparable to those of bouncing embryos. However, embryos categorized as twist‐and‐crumble demonstrated a significant impairment in compaction, blastulation, and expansion compared to the embryos with bouncing, whereas the pregnancy outcomes after blastocyst transfers were not affected by blastomere movement.^73^ Therefore, it is recommended that twist‐and‐crumble embryos should be cultured until the blastocyst stage is reached.

Prolonged blastomere movement (Video S19) detrimentally affects embryo development to the blastocyst stage^73^ and pregnancy outcomes after cleavage‐stage embryo transfers,^72^ regardless of the type of blastomere movement. The duration of blastomere movement does not affect pregnancy outcomes after blastocyst transfer.^73^ If the movement duration during the 2‐cell stage exceeds 0.3 (3–3.3 h when the time during the 2‐cell stage is 10–11 h), these embryos are recommended to be cultured to the blastocyst stage.

#### 3.2.3 Current and future research perspectives

The question of the mechanism underlying aberrant blastomere movements, characterized by twist‐and‐crumble and prolongation occurrence, and if they can be mitigated remains unanswered. In matured oocytes, actomyosin and Arp2/3 complex regulate the actin filaments flow continuously away from the animal cortex.^76^, ^77^, ^78^ This flow retains cortical actin polarization and maintains the chromosomes and spindle at the cortex and in oocytes.^79^, ^80^, ^81^ During fertilization, the distribution of the cytoskeleton, chromosomes, and organelles shifts from asymmetric to symmetric.^65^ The insufficient redistribution of cell components may impact the establishment of cell polarity after the first cell division, which is crucial for embryo development,^82^ generating the aberrant cytoplasmic flow and subsequent blastomere movement. Blastomere movement appears unrelated to patient characteristics, hormonal status, semen quality, or insemination method. Although the extended TI from PN juxtaposition to PNBD and from PNBD to the first cell division reportedly leads to prolonged blastomere movement,^72^ the association between these phenomena and blastomere movement remains unclear. Furthermore, the association between blastomere movement and culture medium has not yet been examined. While there is controversy regarding whether the type of culture medium affects morphokinetics and morphological alteration^83^, ^84^, ^85^, ^86^; it is plausible that the medium may impact blastomere behavior. Molecular studies are required to reveal the mechanism behind aberrant blastomere movement and improve embryonic outcomes by preventing it.

### 3.3 Transzonal projection loss

#### 3.3.1 Typical behavior

Granulosa cells extend transzonal projections, penetrating the zona pellucida to maintain direct contact with oocytes. This enables bidirectional communication between oocytes and granulosa or cumulus cells, which are important structures for oocyte growth.^87^, ^88^, ^89^ Following a luteinizing hormone surge, human chorionic gonadotropin injection, or epidermal growth factor (EGF) treatment, transzonal projections typically disappear via several intermediate steps during oocyte maturation, known as “transzonal projection loss”.^90^, ^91^, ^92^, ^93^, ^94^, ^95^, ^96^, ^97^

#### 3.3.2 Variation and clinical significance

Although there is no study showing that transzonal projections and clinically observed perivitelline threads have the same structure, perivitelline threads are considered to originate from residues of transzonal projections (Video S20).^98^ They are defined as thin filaments extending across the perivitelline space, connecting the zona pellucida with the oolemma or blastomere membrane, observed in 56%–77% of embryos.^98^, ^99^, ^100^ Perivitelline threads are associated with increased fragmentation at first cytokinesis and decreased embryo morphology. However, they are not associated with ploidy status^98^ or pregnancy outcomes.^98^, ^99^ Removing the zona pellucida at the PN stage is one of the methods that prevent perivitelline thread‐associated fragmentation during the early cleavage stage.^101^

#### 3.3.3 Current and future research perspectives

The retraction of transzonal projections is primarily regulated by EGF signaling^95^, ^102^; this suggests that embryos with perivitelline threads may have impaired EGF signaling. As reports regarding perivitelline threads are limited, the functional and clinical relevance of perivitelline threads remains elusive.^100^ The residual degree of perivitelline threads differs among embryos. A minority of zygotes exhibit the perivitelline threads around almost the entire cytoplasm, generating a high degree of fragments that impact the subsequent development. Developing a culture system that stimulates the transzonal projection loss during peri‐insemination would benefit such cases.

### 3.4 Blastomere nucleation

#### 3.4.1 Typical behavior

During zygotic division, the two parental genomes replicate, unite, and segregate into two biparental diploid blastomeres.^69^ The blastomeres form the nucleus, called “blastomere nucleation.” The blastomere nucleation status is defined as the presence or absence of nuclei, and single‐nucleated blastomeres are typically generated.

#### 3.4.2 Variation and clinical significance

Multinucleation is defined as the presence of two or more nuclei of any size in a blastomere (Video S21).^103^ The lagging chromosomes and multipolar segregation can lead to the formation of micronuclei (smaller than 10 μm) around lagging chromosomes or multinucleated daughter cells.^104^, ^105^, ^106^ The incidence of this phenomenon is higher in 2‐cell (40%–65%) than in 4‐cell (15%–30%) embryos.^15^, ^16^, ^107^ The influence of multinucleation on blastocyst formation, pregnancy, and perinatal outcomes is still controversial.^62^, ^108^, ^109^, ^110^, ^111^, ^112^, ^113^, ^114^, ^115^, ^116^, ^117^ Most multinucleated 2‐cell embryos reverse to normal nuclear status when observed at the 4‐cell stage, and persistent multinucleation at the 4‐cell stage is associated with a decreased implantation rate.^107^, ^115^ Multinucleation is suggested to be associated with chromosomal aberrations^36^, ^106^; in fact, most embryos exhibiting the multinucleation at the cleavage stage were diagnosed as mosaics.^118^ However, other studies reported a reduced incidence of multinucleation from the 2‐cell to 4‐cell stages and a similar incidence of multinucleation between the euploid and aneuploid embryos once they developed to the blastocyst stage.^119^ Therefore, most multinucleated embryos likely have the capacity for self‐correction during early cleavage divisions and compaction and can develop into euploid blastocysts, resulting in healthy babies.^32^ Embryos not exhibiting multinucleation during the cleavage stage should be prioritized for the transfer.

#### 3.4.3 Current and future research perspectives

The clinical significance of multinucleation, as previously described, is questionable. Further large‐scale clinical studies are required to determine the effects of multinucleation on IVF outcomes. Additionally, little is known about the origins of the formation of supernumerary nuclei or micronuclei in a blastomere.^32^ Previous studies using somatic cells reported that the knockdown or interference of kinesin family members (Kif), Kif13A, Kif13B, and Kif22, led to the formation of multinucleated cells.^120^, ^121^, ^122^, ^123^ Furthermore, the loss of Kif22‐mediated anaphase chromosome compaction is associated with multinucleation in embryos during the early cleavage stage.^124^, ^125^ Recent studies reported the other possible mechanism, the asynchronicity of PNBD and chromosome capture, which is linked to a different state of chromosome condensation between the two PNs, and multipolar chromosome segregations result in chromosome segregation errors and multinucleation.^36^, ^106^, ^126^ Further studies aimed at identifying the possible causes of asynchronous chromosome condensation and subsequent multinucleation and elucidating the mechanisms underlying self‐correction following multinucleation are of significant interest.

## 4 PERI‐COMPACTION STAGE

### 4.1 Blastomere compaction

#### 4.1.1 Typical behavior

“Blastomere compaction” is the first morphogenetic event that occurs during blastocyst formation and coincides with the first lineage specification decision^127^ (Table 4). This process is typically initiated at the 8–16 cell stage.^128^, ^129^, ^130^ The onset of compaction varies among embryos and is usually confirmed at 74–85 hpi.^15^, ^18^, ^128^ The completion of compaction requires approximately 9–10 h.

**TABLE 4:** Developmental variation during compaction and blastulation.

Phenomena	Variation	Videos	Outcomes
Compaction	Early compaction	Video S22	Blastocyst formation, embryo quality
	Blastomere exclusion	Video S23	Blastocyst formation, embryo quality, live birth
	Blastomere extrusion	Video S24	Blastocyst formation, embryo quality, live birth
	Blastomere exclusion/extrusion	Video S25	Blastocyst formation, embryo quality, live birth
Blastulation/expansion	Blastocyst spontaneous collapse (high‐magnitude)	Video S26	Blastocyst expansion, embryo quality, ploidy, live birth
	Blastocyst spontaneous collapse (low‐magnitude)	Video S27	Blastocyst expansion, embryo quality, ploidy, live birth
	Cytoplasmic strings	Video S28	Blastocyst spontaneous collapse

#### 4.1.2 Variation and clinical significance

Compaction was observed in some embryos during the early cleavage stage. Approximately 10% of embryos initiate compaction before the 8‐cell stage, often categorized as having “early compaction” (Video S22).^130^ The rates of blastocyst formation and good‐quality blastocysts are decreased in embryos showing early compaction.^130^, ^131^ Furthermore, the shortened TI from the 8‐cell stage to compaction onset (<11.5 h) is associated with decreased blastocyst formation and impaired quality.^15^ Therefore, the shortened TI from the 8‐cell stage to compaction onset may also be considered as early compaction. The adverse effects of early compaction on pregnancy outcomes after blastocyst transfer have not been reported. Therefore, embryos should be cultured to the blastocyst stage; however, embryos without early compaction should be prioritized for transfer.

#### 4.1.3 Current and future research perspectives

Early compaction is more likely to be observed in embryos showing a delay in cell division during the cleavage stage^131^ or in embryos derived from young women (<35 years old).^15^ During compaction, outer blastomeres undergo apical‐basal cell polarity acquisition and express protein kinase C‐ζ (PKCζ) at the contact‐free domain. This expression of PKCζ inhibits the Hippo signaling pathway and its nuclear expression effectors, such as Yes‐associated protein (YAP).^15^, ^132^, ^133^, ^134^, ^135^, ^136^ The expression and distribution of these cell polarity markers are crucial for blastomere compaction. Although recent studies have revealed the mechanism of blastomere compaction, it remains unclear why early compaction occurs and how this phenomenon can be avoided. One possible mechanism of early compaction could involve the premature localization of PKCζ at the apical membrane of outer cells. This localization of PKCζ induces nuclear localization of YAP and stimulates the expression of trophectoderm (TE)‐associated genes in outer cells,^134^, ^135^, ^137^ ultimately resulting in early blastomere compaction.

### 4.2 Blastomere inclusion, exclusion, and extrusion during compaction

#### 4.2.1 Typical behavior

Embryos with compaction that include all blastomeres are classified as “fully compacted morula” (or completely compacted morula).^71^, ^138^, ^139^ The incidence of full compaction is approximately 35%–45%, representing the most common compaction pattern.^128^, ^138^, ^139^, ^140^

#### 4.2.2 Variation and clinical significance

During the peri‐compaction period, excluded and extruded cells were identified.^139^ The exclusion of blastomeres from the compaction process at the beginning is termed “blastomere exclusion.” The extrusion of blastomeres from an already compacted morula is termed “blastomere extrusion.” Morulae can be categorized into four groups: fully compacted morulae, partially compacted morulae with excluded cells (Video S23), partially compacted morulae with extruded cells (Video S24), and partially compacted morulae with both excluded and extruded cells (Video S25).^128^ Increased numbers of excluded and extruded blastomeres are associated with decreased blastocyst rate, poor morphology, and impaired live birth rate after blastocyst transfer.^128^, ^139^, ^140^, ^141^ Therefore, fully compacted morulae should be prioritized for transfer. The association between compaction patterns and ploidy status of embryos remains controversial.^139^, ^140^, ^141^, ^142^, ^143^

#### 4.2.3 Current and future research perspectives

Blastomere exclusion is considered a possible self‐correction mechanism aimed at excluding aneuploid cells from mosaic embryos.^61^, ^71^, ^144^ However, further studies are necessary to reveal the function of blastomere exclusion during compaction in human embryo plasticity and self‐correction.^32^

The incidence of blastomere extrusion is increasing in women of advanced maternal age (AMA).^15^ Furthermore, embryos from women of AMA exhibit delayed compaction, decreased PKCζ protein, and a failure of YAP translocation into the nucleus of outer cells of the morula, compared to embryos from young women.^15^ The inhibition, knockdown, or knockout of PKCζ leads to the restricted YAP expression in the cytoplasm,^134^, ^135^, ^145^, ^146^ expression of specific inner cell markers in outer cells of morula, cavitation deficiency, and embryonic arrest at the morula stage.^134^ These findings suggest that insufficient regulation of cell polarity markers, PKCζ, may contribute to insufficient TE differentiation and subsequent increased blastomere extrusion in women with AMA, potentially leading to morula arrest. However, the influence of maternal aging on blastomere compaction, exclusion, and extrusion and the expression of cell polarity markers remain unclear. More basic research is required to shed light on this fascinating area of research.

## 5 BLASTOCYST STAGE

### 5.1 Blastulation (cavitation) and expansion

#### 5.1.1 Typical behavior

Blastocysts are characterized by the formation of a fluid‐filled cavity and an inner cell mass surrounded by the TE, a process known as “blastulation”^147^ (Table 4). As the blastocyst develops, the blastocoel cavity expands, filling the embryo and surpassing its original volume, a stage referred to as “expansion”. Embryos initiate blastulation, primarily regulated by aquaporins and Na+/K+ ATPase isoforms α1, β1, and β3,^148^, ^149^, ^150^, ^151^ at 97–104 hpi and reach the expanded blastocyst stage at 103–120 hpi.^16^, ^152^, ^153^, ^154^

#### 5.1.2 Variation and clinical significance

Human embryos have considerable morphokinetic flexibility, with the ability to achieve blastocyst development occurring between days 4 and 7 (day 4, 0.7%; day 5, 64.0%; day 6, 33.8%; and day 7, 1.6%).^53^ The TI between the initiation of expansion and the achievement of full expansion was longer in days 6 and 7 blastocysts than on days 4 and 5.^53^, ^155^ Increasing times to blastocyst formation is associated with poor embryo quality, decreased euploid rate, and worse pregnancy outcomes, although neonatal outcomes, such as birth length, weight, and malformations, remain unaffected by the developmental speed.^18^, ^19^, ^53^, ^156^, ^157^, ^158^, ^159^ Moreover, developmental speed is associated with pregnancy outcomes, even after single euploid blastocyst transfers.^160^ Therefore, blastocysts from days 4 and 5 should be prioritized for transfer over those from days 6 and 7. However, ending the embryo culture on day 6 results in a relative reduction of 7.3% of patients obtaining euploid blastocysts and a 4.4% decrease in live births.^155^

#### 5.1.3 Current and future research perspectives

The expansion of the blastocoel cavity requires high levels of ATP^150^; however, maternal aging induces decreased mitochondrial function, resulting in reduced ATP and metabolic activity.^49^, ^161^, ^162^ Additionally, maternal aging prolongs the time required for blastocyst expansion.^15^, ^23^, ^154^, ^163^ Therefore, further studies are required to prevent mitochondrial dysfunction‐associated developmental delays. Although developmental speed diverges immediately after fertilization,^53^ the question of whether and how differences among such embryos emerge during fertilization remains entirely unanswered. Determining the factors that affect developmental speed would contribute to understanding the relative impact of intrinsic and extrinsic causes on developmental kinetics and competence, obtaining more blastocysts on days 4 or 5, and improving pregnancy outcomes.

Although not proven, mitotic errors during early embryonic division are considered to result in slower cleavage, longer cell cycles, and subsequently delayed blastocyst formation.^164^, ^165^, ^166^ These embryos have an intrinsic capacity for self‐correction, which may occur through selective apoptosis and reduced proliferation of aneuploid cells.^32^, ^167^, ^168^ A recent study proposed four models for self‐correction: (1) embryonic mortality, (2) aneuploidy rescue, (3) preferential allocation, and (4) clonal depletion. They also reported increased apoptotic levels and impaired differentiation capacity in TE cells of human mosaic and aneuploid embryos.^169^ However, the mechanisms by which embryos detect aneuploid cells and induce apoptosis remain unclear. Revealing the mechanism would provide valuable insights not only into future studies to uncover the cause of differential developmental speed but also into embryo evaluation and selection in the embryology laboratory.

### 5.2 Blastocyst spontaneous collapse/contraction (anomalous behavior)

#### 5.2.1 Typical behavior

“Blastocyst spontaneous collapse” consists of one or more contractions caused by sequential efflux and uptake of blastocoel fluid, probably due to the partial loss and reconstitution of intercellular contacts between TE cells.^170^ Approximately 20%–50% of blastocysts collapse, and the number (1–9 collapses) and degree of collapses vary among embryos (Videos S26 and S27).^153^, ^170^, ^171^, ^172^

#### 5.2.2 Variation and clinical significance

Blastocyst spontaneous collapses and the frequency of these occurrences are associated with degeneration before full expansion, delayed development, reduced morphological quality, and higher rates of aneuploidy.^153^, ^173^ Additionally, blastocyst transfer results in poor pregnancy outcomes.^170^, ^172^, ^173^, ^174^ Blastocysts without spontaneous collapse should be prioritized for transfer.

#### 5.2.3 Current and future research perspectives

The underlying molecular mechanisms and developmental significance of this phenomenon remain poorly understood. The gap junction intercellular communication can be observed from the late cleavage stage and plays an important role in embryo development to the blastocyst stage.^175^, ^176^ A previous study reported that the inhibition of gap junction intercellular communication increases the incidence of blastocyst collapse, suggesting that embryos possessing insufficient gap junction intercellular communication would likely exhibit the collapse after expansion.^177^ Another study showed that a lower number of TE cells per maximum expansion cross‐sectional area correlated with more frequent collapses.^178^ Furthermore, a recent study indicates that slower cell cycles may cause the blastocyst collapse since the tight junctions in the TE would fail to resist the hydrostatic pressure of a progressively increasing blastocoel fluid in these blastocysts.^153^ Further studies are required to elucidate the molecular signals and physical forces governing blastocyst expansion, collapse, and re‐expansion dynamics.^153^ Furthermore, the definition of spontaneous blastocyst collapse reported in previous studies is inconsistent.^153^, ^170^, ^172^, ^173^ Therefore, standardization of the definition of spontaneous blastocyst collapse is clinically needed to improve comparability between future studies.

### 5.3 Cytoplasmic strings

#### 5.3.1 Typical behavior

“Cytoplasmic strings” that bridge the inner cell mass and TE are commonly present in early blastocysts (approximately 55%–85%) and may withdraw as the blastocyst expands (Video S28).^56^, ^179^, ^180^ These strings are suggested to play a role in the direct communication between the mural TE and inner cell mass cells.^181^

#### 5.3.2 Variation and clinical significance

A static observational study reported that their persistence in expanded blastocysts was associated with poor embryo quality, poor media conditions, or breakdown in polarization.^182^ However, recent time‐lapse studies have reported that this morphological feature has no negative impact on pregnancy outcomes. Instead, it is a positive predictor of pregnancy outcomes, although the cytoplasmic strings are associated with increased blastocyst spontaneous collapse.^179^, ^180^, ^183^, ^184^ Consequently, the observation of cytoplasmic strings is currently deemed unnecessary for predicting pregnancy outcomes.

#### 5.3.3 Current and future research perspectives

Although recent studies have suggested that strings positively influence blastocyst expansion and post‐implantation development,^180^, ^183^, ^184^ the clinical and biological significance of cytoplasmic strings has yet to be determined. The vesicle‐like structures moving along cytoplasmic strings can be observed, suggesting that the migration of signaling molecules, including FGRR2 and ErbB3, or the exchange of cytoplasmic material occurs through cytoplasmic strings.^93^, ^180^, ^185^ However, the precise nature of the molecules being transported through cytoplasmic strings and the mechanisms involved remain unknown. Further biological studies are necessary to elucidate how cytoplasmic strings facilitate communication between TE and inner cell mass cells and their potential role in subsequent implantation and development.

## 6 CONCLUSION

The TLT enables us to observe consecutive changes in embryo morphology, serving as biomarkers for embryo assessment. To maximize the clinical benefits of this technology, it is crucial to understand the biological mechanisms and significance of each phenomenon. Therefore, further research on embryonic phenomenology is required in both the clinical and molecular fields. Moreover, in assisted reproductive technology laboratories, several phenomena, such as fertilization deficiency and morula arrest, remain undisclosed. Collaborative work between the clinical and molecular fields and subsequent translational studies are required to advance embryonic outcomes and assessment.

## CONFLICT OF INTEREST STATEMENT

Authors declare no Conflict of Interest for this article.

## HUMAN RIGHTS STATEMENTS AND INFORMED CONSENT

This article does not contain any studies involving human participants that were performed by any of the authors.

## ANIMAL STUDIES

This article does not contain any studies with animal subjects performed by any of the authors.

## Supporting information


Video S1.–S28.
