# Supplementary material for: Developmental perturbation in human embryos: Clinical and biological significance learned from time‐lapse images
Source: Reprod Med Biol. 2024 Jul 9;23(1):e12593. doi: 10.1002/rmb2.12593 (PMC11232294; doi:10.1002/rmb2.12593)
Supplement: Supplementary file 1 — Video S1.–S28. [file RMB2-23-e12593-s001.zip › rmb212593-sup-0029-Legends.docx]

**Supporting information**

**Video 1. Typical behaviors of a human embryo during fertilization and early development.**

**Video 2. Unequal-sized pronuclei in a zygote.**

**Video 3. Delay in pronuclear breakdown.**

**Video 4. Early pronuclear breakdown at 17 h post insemination.**

**Video 5. Non-juxtaposed pronuclear breakdown.**

**Video 6. Asynchronous pronuclear breakdown.**

**Video 7. Failure in the alignment of nucleolus precursor body.**

**Video 8. Nucleolus precursor body alignment in a one-pronuclear zygote.**

**Video 9. Nucleolus precursor body alignment in a non-juxtaposed pronuclear zygote.**

**Video 10. Absence of a cytoplasmic halo in a zygote.**

**Video 11. Prolonged cytoplasmic halo in a zygote.**

**Video 12. Unstable cytoplasmic halo in a zygote.** Cited from Ezoe et al. [45].

**Video 13. Direct (trichotomous) cleavage at the first cell division (duration of 2-cell stage = 0 h).**

**Video 14. Rapid cleavage at the first cell division (duration of 2-cell stage >0 h and < 5 h).**

**Video 15. Asymmetric division at the first cell division.**

**Video 16. Reverse cleavage (cell fusion) after the first cell division.**

**Video 17. Blastomere wobbling after the first cell division.**

**Video 18. Twist-and-crumble after the first cell division.**

**Video 19. Prolonged blastomere movement after the first cell division.**

**Video 20. Perivitelline threads during the first cell division.**

**Video 21. Multinucleation during the cleavage stage.**

**Video 22. An early compacted embryo.**

**Video 23. Partially compacted morulae with excluded cells.**

**Video 24. Partially compacted morulae with extruded cells.**

**Video 25. Partially compacted morulae with both excluded and extruded cells.**

**Video 26. High**-**magnitude blastocyst spontaneous collapse.**

**Video 27. Low**-**magnitude blastocyst spontaneous collapse.**

**Video 28. Cytoplasmic strings that bridge the inner cell mass and trophectoderm.**
